# The role of transcutaneous auricular vagus nerve stimulation in chronic pain: from neurobiological mechanisms to clinical applications

**DOI:** 10.3389/fpain.2026.1733445

**Published:** 2026-01-29

**Authors:** Jing Zhang, Yang Zhang, Jingxue Zhao, Jifei Sun, Xiaoxu Zhang

**Affiliations:** 1 Department of Pain Management, Beijing Fengtai You'anmen Hospital, Beijing, China; 2 Department of Traditional Chinese Medicine, Beijing Genertec Aerospace Hospital, Beijing, China; 3 China Academy of Chinese Medical Sciences, Guang'anmen Hospital, Beijing, China; 4 Department of Encephalopathy, Beijing Hospital of Traditional Chinese Medicine Shunyi Hospital, Beijing, China

**Keywords:** chronic pain, neuromodulation, pain mechanisms, review, transcutaneous auricular vagus nerve stimulation, anxiety

## Abstract

Chronic pain is a prevalent health issue with high disability rates, and traditional pharmacological treatments often come with limitations such as dependency and side effects. Transcutaneous auricular vagus nerve stimulation (taVNS), as an emerging non-invasive neuromodulation technique, has demonstrated broad application prospects in chronic pain management in recent years. This systematic review examines the clinical efficacy of taVNS across multiple chronic pain conditions, including neuropathic pain, autoimmune disease-related pain, gastrointestinal pain, and musculoskeletal pain. It also delves into its neurobiological mechanisms, primarily involving activation of central descending pain control pathways, modulation of cholinergic anti-inflammatory pathways, balancing autonomic nervous system function, reshaping functional connectivity in brain networks, regulating neurotransmitter and neuropeptide balance, and inhibiting peripheral and central sensitization processes. Despite ongoing challenges in parameter standardization, in-depth mechanism elucidation, and personalized treatment strategies, taVNS offers an innovative therapeutic approach for chronic pain patients due to its favorable safety profile, tolerability, and multi-target regulatory advantages. Future large-scale clinical studies and multidisciplinary collaboration are needed to further advance the precision application of taVNS within comprehensive pain management systems.

## 1 Introduction

Pain is an unpleasant sensory and emotional experience associated with actual or potential tissue damage (1). Chronic pain typically refers to pain lasting longer than 3–6 months, affecting approximately 20%–30% of adults worldwide (2). It not only frequently co-occurs with emotional disorders such as depression and anxiety but also impairs cognitive function (3). The economic burden of chronic pain now exceeds that of cancer and cardiovascular disease combined, affecting over 1.5 billion people globally (4, 5). As one of the most common reasons for seeking medical care, chronic pain severely impairs patients’ quality of life and work performance.

Currently, the management of chronic pain primarily relies on medication, but this approach has significant limitations (6). Nonsteroidal anti-inflammatory drugs may damage the gastrointestinal tract and kidneys, while antiepileptic drugs often cause drowsiness and dizziness. Opioids, though potent, carry risks of addiction and respiratory depression (7). These medications often address symptoms rather than the root cause, and long-term use can lead to dependence and cumulative side effects. Interventional therapies like nerve blocks offer precision and speed but provide only short-term relief, carrying risks of infection or nerve damage (8). Psychological approaches such as cognitive behavioral therapy can reshape pain perception, yet they are often overlooked because they cannot eliminate physical pain sensations (9). Their effectiveness also depends heavily on patient acceptance and cooperation. Consequently, there is an urgent clinical need to develop novel treatment strategies that combine sustained efficacy with favorable safety profiles.

## 2 Neuropathic pathways of chronic pain

**Figure 1:**
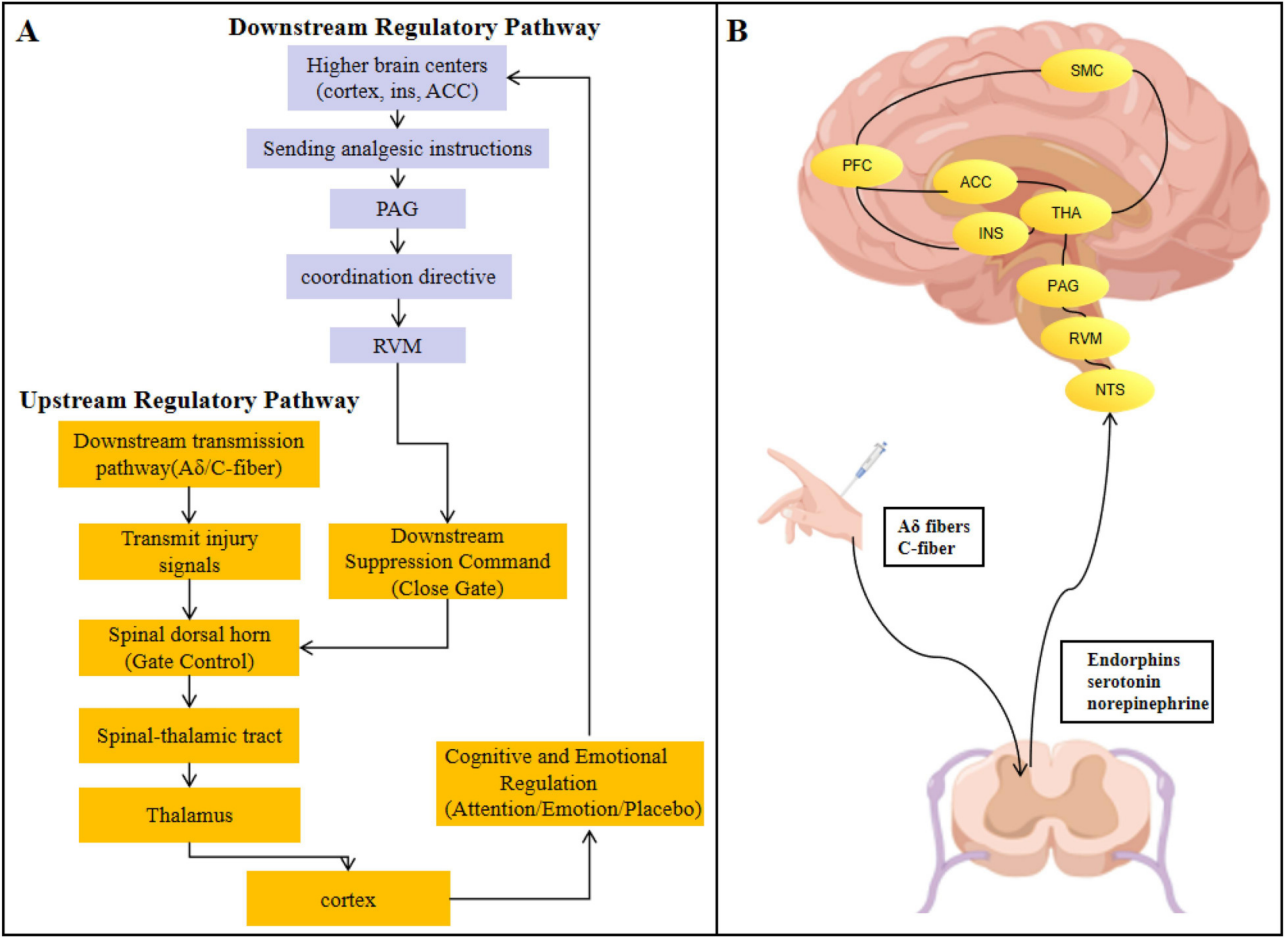
Specific transmission pathways of chronic pain from peripheral to central regions. **(A)** Flowchart illustrating the directive pathways of pain transmission; **(B)** Key brain regions involved in pain processing. INS, insular cortex; ACC, anterior cingulate cortex; PFC, prefrontal cortex; SMC, somatosensory motor cortex; THA, thalamus; PAG, periaqueductal gray; RVM, rostral ventromedial medulla; NTS, nucleus tractus solitarius.

The pain pathway begins when various noxious stimuli activate peripheral nociceptors distributed throughout the skin, muscles, viscera, and other tissues (10, 11). These specialized nerve endings convert harmful stimuli into electrical signals (Figure 1). These signals are transmitted to the central nervous system via two distinct types of primary afferent nerve fibers: larger-diameter, myelinated A*δ* fibers convey rapid, precisely localized “primary pain” as an early warning signal. while smaller, unmyelinated C fibers transmit slow, diffuse “second pain,” often manifesting as burning sensations or dull aches, closely associated with chronic discomfort (12, 13). These afferent fibers primarily terminate in specific laminae of the dorsal horn of the spinal cord, forming complex synaptic connections with secondary neurons (14, 15). This represents the first critical node for pain modulation, where the renowned “gate control theory” manifests. Descending pain modulation systems originating from the brain release substances such as endorphins, serotonin, and norepinephrine at this site (16). Through presynaptic and postsynaptic mechanisms, they selectively inhibit or facilitate the transmission of pain signals, acting like a “gate.” This represents the crucial structural basis for endogenous analgesia and chronic pain sensitization (17).

However, the gate control theory only explains the preliminary modulation of nociceptive signals at the spinal dorsal horn level and is insufficient to fully elaborate the complex pathophysiological nature of neuropathic chronic pain. Neuropathic pain arises from injury or disease of the somatosensory nervous system, and its core characteristics involve long-term maladaptive changes, including peripheral sensitization, central sensitization, and structural and functional reorganization of spinal and supraspinal neural circuits (2, 18, 19). At the level of peripheral sensitization, local inflammation or nerve injury leads to increased excitability and decreased threshold of nociceptors, resulting in hyperalgesia (18). During this process, damaged nerve endings exhibit ectopic discharge, and the expression and function of voltage-gated sodium channels and calcium channels are abnormal, enhancing the signal transmission ability of peripheral nerves (20, 21). In addition, inflammatory factors released by the infiltration of immune cells at the injury site further activate nociceptors, forming a “inflammation-pain” vicious cycle that continuously amplifies peripheral pain signals (22, 23).

At the level of central sensitization, persistent peripheral injury signals induce plastic changes in spinal and cerebral pain pathways (24). The synaptic transmission of spinal dorsal horn neurons is enhanced, manifested as the long-term potentiation effect triggered by the activation of glutamatergic receptors, which expands the response field of neurons and causes pain responses to non-noxious stimuli (19). Meanwhile, the descending inhibitory system centered on the locus coeruleus and raphe nucleus is weakened, and the release of inhibitory neurotransmitter is reduced, further exacerbating the central amplification of pain signals (17, 25). At the supraspinal level, the abnormal increase in neuronal excitability in brain regions such as the thalamus, anterior cingulate cortex, and insular cortex participates in the remodeling of the affective-motivational dimension and cognitive evaluation process of pain, making the pain experience more persistent and intense (26–28).

In addition, neuropathic pain also involves deep mechanisms such as structural and functional reorganization of neural circuits, regulation of gene expression, and neuroimmune interactions (18, 23). Long-term pain stimulation leads to increased dendritic spine density and synaptic structure remodeling of spinal dorsal horn neurons, as well as abnormal expression of pain-related genes (21). Neuroimmune crosstalk plays a key role in this process: activated microglia and astrocytes in the central nervous system release a large number of pro-inflammatory factors and chemokines, which further aggravate neuroinflammation and synaptic plasticity abnormalities, forming the pathological basis of chronic pain (22, 29). These multi-level pathological changes collectively constitute a complex mechanism network of neuropathic pain, which is far beyond the explanatory scope of the gate control theory.

After preliminary integration and modulation in the dorsal horn of the spinal cord, pain signals are primarily received by spinal cord interneurons and transmitted upward via the spinothalamic tract formed by their axons (30). This critical pathway is responsible for conveying information about the location, intensity, and nature of pain to higher-level centers. Additionally, fibers conveying slow pain and emotional components also project through pathways such as the ancient spinoreticular tract (31). The thalamus, serving as the central relay station for all sensory information, functions as the “master control room” in this process. Its specific nuclei perform initial processing and classification of pain signals, then precisely distribute them to distinct regions of the cerebral cortex. This prepares the groundwork for subsequent refined perception and emotional experience (32).

The ultimate perception and experience of pain is a complex process accomplished through the coordinated effort of distributed brain networks (33). Within the cerebral cortex, distinct brain regions form a specialized processing pipeline: the primary and secondary somatosensory cortices primarily analyze the sensory discrimination dimensions of pain, while the anterior cingulate cortex and insula deeply process its affective-motivational dimensions (26, 28). Together, they endow pain with its inherently unpleasant and aversive qualities, triggering associated autonomic responses—considered the core mechanism driving the distress of pain and motivating avoidance behaviors. Higher-order prefrontal cortex regions oversee cognitive evaluation, contextual understanding, attentional allocation, and anticipatory regulation of pain (34). This feedback inhibition of spinal dorsal horn activity by the cerebral cortex provides the neurophysiological basis for psychological factors to significantly influence pain experience (25).

In summary, the essence of chronic pain lies in the pathological disruption of the body's homeostatic regulatory mechanisms governing the perception and transmission of nociceptive signals during acute pain states. Effectively curbing the progression of pain chronicity and improving clinical outcomes hinges on restoring the normal function of this regulatory network. Existing research indicates that the core objective of multiple clinical intervention strategies is to restore the overall regulatory balance of pain pathways (24–35). By targeting and modulating functional abnormalities at various points within the neural transmission pathways, these approaches not only promote the reconstruction of the nervous system's intrinsic homeostasis but also restore its physiological functions, ultimately significantly enhancing patients’ quality of life.

## 3 The origin and development of transcutaneous auricular vagus nerve stimulation

The theoretical framework of taVNS represents a convergence of Eastern and Western medical knowledge in neuromodulation, with historical roots spanning over two millennia. Its theoretical origins can be traced to the ancient Chinese medical classic Yellow Emperor's Inner Canon, which documented early understanding of functional correlations between the auricle and the body's meridian systems and visceral organs, establishing the fundamental principles of auricular therapy (36). In the 1950s, French physician Paul Nogier systematically developed the “inverted fetus” somatotopic model and created detailed auricular topographic maps, recognized as a foundational contribution to modern auricular medicine (37). Contemporary neuroanatomical research has further validated that the cymba conchae and cavum conchae regions receive specific innervation from the auricular branch of the vagus nerve (ABVN) (38, 39). This anatomical region has been identified as the exclusive cutaneous distribution area of the vagus nerve, providing the scientific basis for non-invasive vagal modulation through transauricular stimulation.

In parallel to the development of taVNS, another non-invasive neuromodulation approach has emerged: non-invasive vagus nerve stimulation (VNS) applied to the right anterior cervical region overlying the carotid artery (40). This technique delivers mild transcutaneous electrical stimulation to activate vagal afferent fibers via the cervical branch, offering a non-surgical alternative pathway for vagal modulation (41). Studies have shown that cervical non-invasive VNS can effectively modulate autonomic tone, reduce sympathetic outflow, and exert anti-inflammatory and analgesic effects, with established applications in migraine, cluster headache, and other pain conditions (42, 43). Although targeting a different anatomical site, cervical non-invasive VNS shares overlapping mechanisms with taVNS, such as activation of the nucleus tractus solitarius and modulation of central pain pathways (44). The coexistence of these two non-invasive VNS modalities enriches the toolbox for neuromodulation-based pain management and provides clinicians with flexible options tailored to individual anatomical and physiological characteristics.

Prior to the emergence of taVNS, VNS was predominantly achieved through implanted devices. The year 1997 marked a significant milestone when the U.S. Food and Drug Administration granted approval for implantable vagus nerve stimulation in managing drug-resistant epilepsy, establishing invasive VNS as a validated clinical intervention (45, 46). However, constraints including surgical invasiveness, substantial costs, and implantation-related complications limited its widespread clinical implementation (47). To address these challenges, researchers developed taVNS as an innovative alternative, enabling safe, accessible, and targeted non-invasive neuromodulation through transcutaneous electrical stimulation of auricular vagal territories using specific stimulation parameters (42, 48).

With advancing clinical adoption, the mechanistic underpinnings and therapeutic applications of taVNS have been progressively elucidated. Neurophysiological studies demonstrate that taVNS activates ABVN fibers, conducting signals to the nucleus tractus solitarius in the brainstem, with subsequent propagation through the reticular activation system to key neuromodulatory regions including the locus coeruleus, prefrontal cortex, and limbic circuitry (49, 50). This central pathway mediates the regulation of critical neurotransmitters including norepinephrine, gamma-aminobutyric acid (GABA), and serotonin (51). Clinically, the application spectrum of taVNS has expanded from its initial use in epilepsy to include depression, anxiety disorders, insomnia, and cognitive disorders (52). Particularly noteworthy is its emerging role in chronic pain management. Recent evidence confirms that taVNS not only significantly reduces pain intensity but also ameliorates comorbid negative affect and sleep disturbances, leading to comprehensive improvement in quality of life (53).

Thus, taVNS offers a potential non-invasive alternative to the invasive VNS that has been in use for the past two decades. By stimulating the auricular branch of the vagus nerve transcutaneously, it retains the core neuromodulatory principle of traditional VNS while circumventing the need for surgical implantation (45, 47). This approach directly addresses key limitations of invasive VNS, such as surgical risks, high costs, and device-related complications (48). Compared with conventional pharmacotherapy, taVNS offers distinctive advantages including non-invasiveness, favorable safety profile, and operational practicality, positioning it as a promising therapeutic alternative in chronic pain management (54).

## 4 Common types of chronic pain treated with taVNS

**Figure 2:**
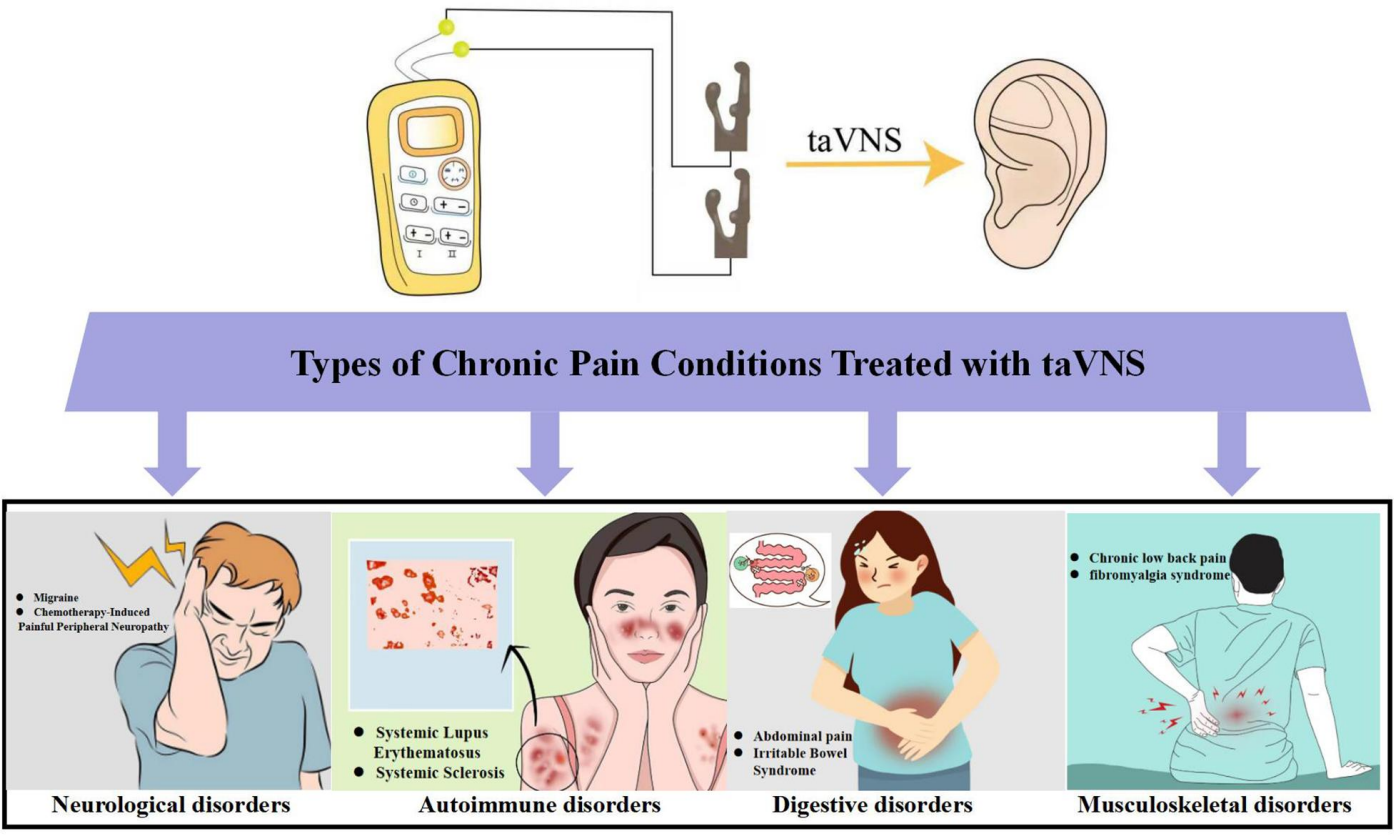
This diagram illustrates the application of taVNS in treating various chronic pain conditions, primarily encompassing those originating from neurological (migraine, chemotherapy-induced peripheral neuropathy), autoimmune (systemic lupus erythematosus, systemic sclerosis), gastrointestinal (irritable bowel syndrome,persistent abdominal pain), and musculoskeletal (chronic low back pain, fibromyalgia syndrome) disorders.

Recent years have witnessed a notable expansion in the body of research exploring the application of taVNS for chronic pain. Substantial evidence indicates that taVNS demonstrates promising efficacy in alleviating various chronic pain conditions stemming from neurological, autoimmune, gastrointestinal, and musculoskeletal disorders (Figure 2 and Table 1). Current research predominantly focuses on pain associated with neurological disorders, while exploration of pain in autoimmune diseases remains relatively limited. This disparity highlights the vast unexplored potential and inherent advantages of taVNS within the field of chronic pain management.

**Table 1:** Clinical studies of taVNS in chronic pain.

Author (year)	Subjects	*n*	Site	taVNS parameter	Main results
Straube et al. (2015) (47)	Chronic migraine	46 (22 for 1 Hz, 24 for 25 Hz)	Left concha	Frequency: 1/25 Hz Pulse width: 250 µs Intensity: 0–2 mA Duration: 4 weeks	1 Hz taVNS significantly reduced headache frequency and disability with a favorable safety profile, demonstrating superiority over 25 Hz stimulation.
Aranow et al. (2020) (61)	SLE patients with musculoskeletal pain	18 (12 taVNS, 6 sham)	taVNS: Left cymba concha Sham: Earlobe	Frequency: 30 Hz Pulse width: 300 µs Duration: 4 days	TaVNS significantly reduced pain, fatigue, tender/swollen joints, and substance P levels.
Zhang et al. (2020) (48)	Migraine without aura	59 (33 taVNS, 26 sham)	taVNS:Left cymba concha Sham:Left tail of the helix	Frequency: 1 Hz Pulse width: 0.2 ms Intensity: 1.5–5 mA Duration: 4 weeks	TaVNS alleviated migraine symptoms and modulated thalamocortical connectivity, with changes correlating to clinical improvement.
Kutlu et al. (2020) (78)	Fibromyalgia syndrome	27 taVNS + Exercise, 25 Exercise only	Bilateral inner & rear tragus and concha	Frequency: 10 Hz Pulse width: <500 µs Duration: 4 weeks	TaVNS provided superior benefits over exercise alone in physical function, social function, and pain-related quality of life.
Luo et al. (2020) (49)	Migraine without aura	27 (crossover)	taVNS: Left cymba concha Sham: Left scapha	Frequency: 1 Hz Pulse width: 0.2 ms Duration: 8 minutes	TaVNS decreased functional connectivity between the amygdala and pain-related regions. Left amygdala–right SMA connectivity correlated with migraine attack frequency.
Cao et al. (2021) (51)	Migraine without aura	24 (single-blind, crossover)	Left cymba conchae	Frequency: 1/20 Hz Intensity: 0–4 mA Duration: 8 minutes	1 Hz taVNS enhanced PAG connectivity with key pain-processing regions and was superior to 20 Hz in modulating descending pain pathways. Increased PAG–MCC connectivity correlated with fewer migraine attacks.
Shi et al. (2021) (70)	Constipation-predominant IBS	42 (21 taVNS, 21 sham)	Bilateral auricular cymba concha	Frequency: 25 Hz Pulse width: 0.5 ms Intensity: 0–2 mA Duration: 4 weeks	TaVNS effectively relieved constipation, pain, and psychological distress, associated with modulated rectal sensitivity, inflammation, serotonin, and vagal tone.
Paccione et al. (2022) (80)	Fibromyalgia	116 (28 tVNS vs 29 Sham tVNS vs 29 MDB vs 30 Sham MDB)	tVNS: Left cymba conchae Sham tVNS: Left earlobe	Duration: 14 days	All groups showed significant improvement in overall fibromyalgia severity, but no significant between-group differences were found in average pain intensity.
Feng et al. (2022) (52)	Migraine without aura	60	Left cymba concha	Frequency: 1 Hz Pulse width: 0.2 ms Duration: 4 weeks	Baseline fALFF abnormalities in key brain regions were modulated after taVNS, effectively predicting its therapeutic efficacy using an SVR model.
Abdel-Baset et al. (2023) (81)	Fibromyalgia	99 (33 tVNS; 33 PNE; 33 tVNS combined PNE)	Left cymba concha	Frequency: 25 Hz Duration: 2 weeks	All groups improved, but combined tVNS and PNE was superior, showing the greatest improvement. The therapy was safe and well-tolerated.
Bellocchi et al. (2023) (65)	Systemic sclerosis patients with chronic pain	35 (crossover)	Left cymba concha	taVNS: 25 Hz, 250 µs, 4 days Sham: 1 Hz, 250 µs, Duration:2 weeks	TaVNS significantly reduced chronic pain and plasma IL-6 levels compared to control, was safe, but did not improve quality of life or autonomic function.
Huang et al. (2023) (50)	Migraine	70 randomized (59 completed: 33 taVNS, 26 sham)	Real: Left cymba concha Sham: Left ear helix	Frequency: 1 Hz Duration: 4 weeks	TaVNS significantly reduced migraine days and pain intensity, potentially by modulating brainstem-limbic functional connectivity, including a key RN-putamen pathway correlated with clinical improvement.
Rao et al. (2023) (53)	Migraine without aura	35 patients, 38 HCs	Left cymba concha	Frequency: 1 Hz Pulse width: 0.2 ms Duration: 4 weeks	TaVNS alleviated symptoms by modulating brain networks, altering centrality in key regions and reconfiguring ITG and cerebellar connectivity. ITG–IPL change correlated with headache improvement.
Figueiredo et al. (2024) (75)	Chronic low back pain	30	Left cymba concha	Frequency: 25 Hz Pulse width: 50 µs Duration: 3 months	TaVNS significantly reduced pain, disability, catastrophizing, and improved quality of life. Over half of the patients achieved clinically meaningful pain relief.
Yang et al. (2024) (57)	Chemotherapy-induced painful peripheral neuropathy	24 (14 taVNS vs 10 sham)	Bilateral cymba conchae and antihelix	Frequency: 20 Hz Pulse width: 0.2 ms ± 30% Intensity: 4–6 mA Duration: 12 days	TaVNS provided short-term relief from subjective pain and improved sleep and mental quality of life, suggesting a central nervous system mechanism of action.
Cai et al. (2025) (71)	Persistent abdominal pain	31	Left cymba conchae	Frequency: 20 Hz Duration: 20 days	TaVNS significantly reduced pain and modulated functional connectivity, notably decreasing connectivity between salience and somatomotor networks.
Li et al. (2025) (76)	Chronic low back pain	51 (25 taVNS, 26 tGANS)	taVNS: Auricular concha cymba/cavum tGANS: Earlobe	Frequency: 20 Hz Duration: 4 weeks	TaVNS augments PAG-limbic and VTA-reward connectivity, whereas tGANS diminishes PAG-limbic connectivity while promoting VTA integration with limbic and sensorimotor regions.

taVNS, transcutaneous auricular vagus nerve stimulation; SLE, systemic lupus erythematosus; SMA, supplementary motor area; PAG, periaqueductal gray; MCC, middle cingulate cortex; IBS, irritable bowel syndrome; SVR, support vector regression; PNE, pain neuroscience education; RN, raphe nucleus; ITG, inferior temporal gyrus; IPL, inferior parietal lobule; VTA, ventral tegmental area; tGANS, transcutaneous greater auricular nerve stimulation.

### 4.1 TaVNS for neuropathic pain disorders

#### 4.1.1 TaVNS for migraine

Migraine is primarily characterized by severe, throbbing headaches on one or both sides of the head, often accompanied by symptoms of autonomic nervous system dysfunction such as nausea, vomiting, and photophobia. It is clinically defined by recurrent episodes and a chronic, difficult-to-treat nature (55). Epidemiological data indicate a global annual prevalence of approximately 14%, with a higher incidence among women (56). TaVNS, a non-invasive neuromodulation technique, has demonstrated promising applications in migraine prevention and treatment in recent years. The first landmark randomized controlled trial in this field was completed by Straube et al. in 2015 (57). This study enrolled 46 chronic migraine patients using a cross-over design comparing different frequencies. Results demonstrated that 1 Hz taVNS significantly outperformed 25 Hz stimulation in reducing headache frequency and improving functional disability, with good safety profiles. This research established the efficacy foundation for low-frequency stimulation in subsequent taVNS applications for migraine and preliminarily validated its clinical potential.

As research progressed, taVNS methodologies for migraine gradually standardized, significantly enhancing comparability and evidence strength. In study design, randomized, sham-controlled trials became the standard paradigm, replacing earlier cross-frequency comparison approaches (58–60). Sham stimulation sites are typically selected in areas with sparse vagus nerve distribution, such as the left auricle or helix, effectively ensuring blinding by mimicking the somatosensory effects of genuine stimulation.

Regarding stimulation parameters, 1 Hz low-frequency stimulation has become the mainstream choice. Multiple studies indicate its superiority over 20 Hz (61) or 25 Hz (57) stimulation in modulating brain functional connectivity (FC) and improving clinical symptoms, suggesting its mechanism may rely more on slow, long-term neuroplasticity regulation. Pulse width parameters also show convergence, typically set within the 200–250 µs range (57–59, 62, 63). While specific intensity ranges vary, the principle of “individualized sensory threshold titration” is universally followed—adjusting to a level producing distinct tingling without causing pain—thus ensuring efficacy while prioritizing tolerability and safety. Regarding treatment duration, a 4-week total course is widely adopted as the standard period for evaluating clinical efficacy (57, 58, 60, 62, 63), deemed sufficient to induce and observe stable clinical and neurophysiological changes.

In summary, the fundamental paradigm for taVNS in migraine research has been preliminarily established, applying 1 Hz stimulation with 200–250 µs pulse width and individualized intensity to the left concha region for 4 weeks, rigorously controlled against sham stimulation in non-vagal areas. This standardized protocol provides a crucial foundation for comparative and integrated subsequent studies, meta-analysis, and clinical translation. Neuroimaging evidence further suggests that the therapeutic effects of taVNS may stem from its multi-node, network-based regulation of the brain's pain networks and descending pain modulation systems.

#### 4.1.2 TaVNS for chemotherapy-induced painful peripheral neuropathy

Chemotherapy-induced peripheral neuropathy (CIPN) is one of the common adverse reactions in cancer patients following intravenous chemotherapy administration, primarily triggered by highly neurotoxic chemotherapeutic agents (64). Typical clinical manifestations of CIPN include stocking-and-glove distribution sensory abnormalities, cold sensitivity, numbness or pain in the extremities, and muscle cramps. Symptom severity typically peaks on days 2–3 after drug administration and progressively worsens during subsequent chemotherapy cycles (65, 66), significantly impacting treatment progression and quality of life.

Yang et al. (67) divided CIPN patients into a 14-patient taVNS group and a 13-patient sham stimulation group. Results demonstrated that taVNS significantly alleviated CIPN-related neuropathic pain with effects lasting up to 30 days, while also improving sleep quality and emotional state. This study first demonstrated the favorable short-term analgesic efficacy and safety of taVNS. However, no significant changes in peripheral nerve function or inflammatory markers were observed, suggesting its mechanism may primarily involve central regulation rather than peripheral nerve repair. This indicates taVNS holds promise as a novel clinical intervention strategy for chemotherapy-induced peripheral neuropathy pain.

### 4.2 TaVNS for autoimmune disorders

#### 4.2.1 TaVNS for systemic lupus erythematosus

Systemic lupus erythematosus (SLE) is a common autoimmune disease, with up to 95% of patients suffering from musculoskeletal pain that severely impairs their quality of life (68, 69). Based on evidence that VNS suppresses inflammatory mediator release and demonstrates efficacy in animal models (70), Aranow et al. (71) conducted a double-blind, randomized controlled trial to evaluate the efficacy of taVNS for musculoskeletal pain in SLE patients. Results showed that the 4-day taVNS treatment was well tolerated and significantly superior to sham stimulation in alleviating pain and fatigue, with effects persisting through day 12. Additionally, both patient and physician global assessments and joint counts improved, correlating with cumulative stimulation dose. Mechanistically, while significant reductions in plasma substance P were observed, other inflammatory mediators showed no significant changes. This suggests that the analgesic and anti-fatigue effects of taVNS may originate from other neuroimmune mechanisms.

#### 4.2.2 TaVNS for systemic sclerosis

Systemic sclerosis (SSc) is a complex autoimmune disease primarily characterized by skin and multi-organ fibrosis, along with vascular lesions (72). Pain is a common clinical symptom affecting approximately 83% of patients, with pain severity significantly correlated with impaired daily functioning and reduced quality of life (73, 74). To explore novel effective pain management strategies, Bellocchi et al. (75) conducted a randomized crossover trial evaluating the efficacy of taVNS as an adjunctive non-invasive neuromodulation therapy for SSc patients. Results demonstrated that a 4-week taVNS intervention significantly reduced pain intensity and downregulated levels of the inflammatory cytokine IL-6 compared to the control group. Although no significant intergroup differences were observed in pain interference, heart rate variability, or other quality-of-life measures, the study confirmed taVNS as a safe and effective noninvasive analgesic method, showing potential application prospects in pain management for autoimmune diseases.

### 4.3 TaVNS for digestive disorders

Irritable bowel syndrome (IBS) is a common functional gastrointestinal disorder characterized primarily by abdominal pain or discomfort accompanied by changes in bowel habits (76). Its prevalence has been rising in recent years, with epidemiological studies indicating that approximately 7%–21% of the global population experiences IBS symptoms (77, 78). Although not directly life-threatening, this condition significantly impairs patients’ quality of life and imposes a substantial healthcare burden (79).

To investigate the efficacy and mechanisms of taVNS in alleviating abdominal pain and constipation in patients with constipation-predominant IBS (IBS-C), Shi et al. (80) conducted a randomized controlled trial. Forty-two IBS-C patients were assigned to either the taVNS group or a sham stimulation group for a 4-week intervention. Results demonstrated that taVNS not only significantly alleviated abdominal pain and increased weekly complete spontaneous bowel movements but also effectively improved patients’ quality of life and depression levels. Another study involving patients with persistent abdominal pain lasting over 6 months further indicated that a 20-day taVNS treatment significantly reduced abdominal pain and enhanced quality of life. Combined with electroencephalography technology, it enabled early prediction of treatment efficacy (81). These studies confirm that taVNS, as a non-invasive, highly safe, and well-tolerated neuromodulation technique, offers a novel treatment option for patients with gastrointestinal pain. It expands the application prospects of non-pharmacological therapies in the comprehensive management of chronic pain.

### 4.4 TaVNS for musculoskeletal disorders

#### 4.4.1 TaVNS for chronic low back pain

Chronic low back pain (CLBP) is one of the most common types of chronic pain, affecting a large global population and representing a major disease burden contributing to disability (82). Statistics indicate that CLBP accounts for 7.7% of all disability-adjusted life years worldwide, making it the leading cause of disability (83). Furthermore, due to prolonged disease duration and persistent pain, approximately 20% of patients with acute low back pain ultimately progress to CLBP annually (84). Despite the availability of various treatment modalities, such as medication, physical therapy, and surgery, pain relief remains limited for most patients (85). Against this backdrop, taVNS, an emerging neuromodulation technique, has garnered increasing attention in recent years. A prospective pilot study by Figueiredo et al. (85) evaluated the feasibility, safety, and preliminary efficacy of taVNS in CLBP patients. The study enrolled 30 patients who received daily 30-minute taVNS interventions over three months. Results demonstrated significant reductions in Visual Analogue Scale (VAS) scores at both 1 and 3 months, alongside marked improvements in quality of life and pain catastrophizing scores. Treatment adherence was high, with no serious adverse events reported, suggesting potential clinical utility for taVNS in CLBP management. However, randomized controlled trials are needed to further validate its efficacy. Separately, Li et al. (86) used functional magnetic resonance imaging (fMRI) to investigate taVNS's effects on FC within the descending pain control system and reward network in CLBP patients. This randomized controlled trial divided 70 patients into a taVNS group and a trigeminal auricular nerve stimulation control group for 4 weeks of treatment. The study found significant improvements in pain intensity and functional impairment in both groups. Further FC analysis revealed that taVNS primarily enhanced FC between the periventricular gray matter of the midbrain and limbic system regions such as the amygdala and anterior cingulate cortex, providing preliminary imaging evidence for understanding the neural mechanisms underlying taVNS's efficacy in alleviating CLBP.

#### 4.4.2 TaVNS for fibromyalgia syndrome

Fibromyalgia syndrome (FMS) is a chronic condition with an etiology that remains incompletely understood, primarily characterized by widespread pain throughout the body and multiple physical discomforts (87). This syndrome predominantly affects women, with peak incidence occurring between the ages of 30 and 50 (88). Its prevalence in the general population ranges from approximately 2.9%–4.7% (89). Although FMS predominantly affects adults, it can also occur in children and the elderly. Current treatment outcomes for fibromyalgia remain suboptimal, prompting researchers to explore neuromodulation therapies. Among these, taVNS has garnered significant attention as an emerging intervention. Kutlu et al. (88) conducted a randomized trial comparing exercise alone versus exercise combined with taVNS in 60 female FMs patients. While both groups showed significant improvements in pain, depression, anxiety, and quality of life, the addition of taVNS did not yield statistically significant benefits over exercise alone. This suggests that taVNS may offer limited incremental benefit when combined with structured exercise programs.

In contrast, Paccione et al. (90) compared active taVNS, sham taVNS, and meditative diaphragmatic breathing in 116 FMs patients. They found no significant changes in heart rate variability (HRV) or average pain intensity across groups, though some improvements in FMs severity and current pain were noted. The study highlighted challenges in measuring vagal tone via short-term HRV and raised questions about the specificity of taVNS effects in FMs. More recently, Abdel-Baset et al. (91) evaluated taVNS alongside pain neuroscience education (PNE) in 99 FMs patients. Their results indicated that the combination of taVNS and PNE led to superior outcomes in pain, catastrophizing, anxiety, and functional impact compared to either intervention alone. This supports the potential of taVNS as part of a multimodal therapeutic approach. Collectively, these studies underscore the promise of taVNS as a non-invasive neuromodulatory tool for FMs, while also highlighting the need for optimized stimulation parameters, longer treatment durations, and combination strategies to maximize therapeutic outcomes.

## 5 Potential mechanisms of action of taVNS in chronic pain management

**Figure 3:**
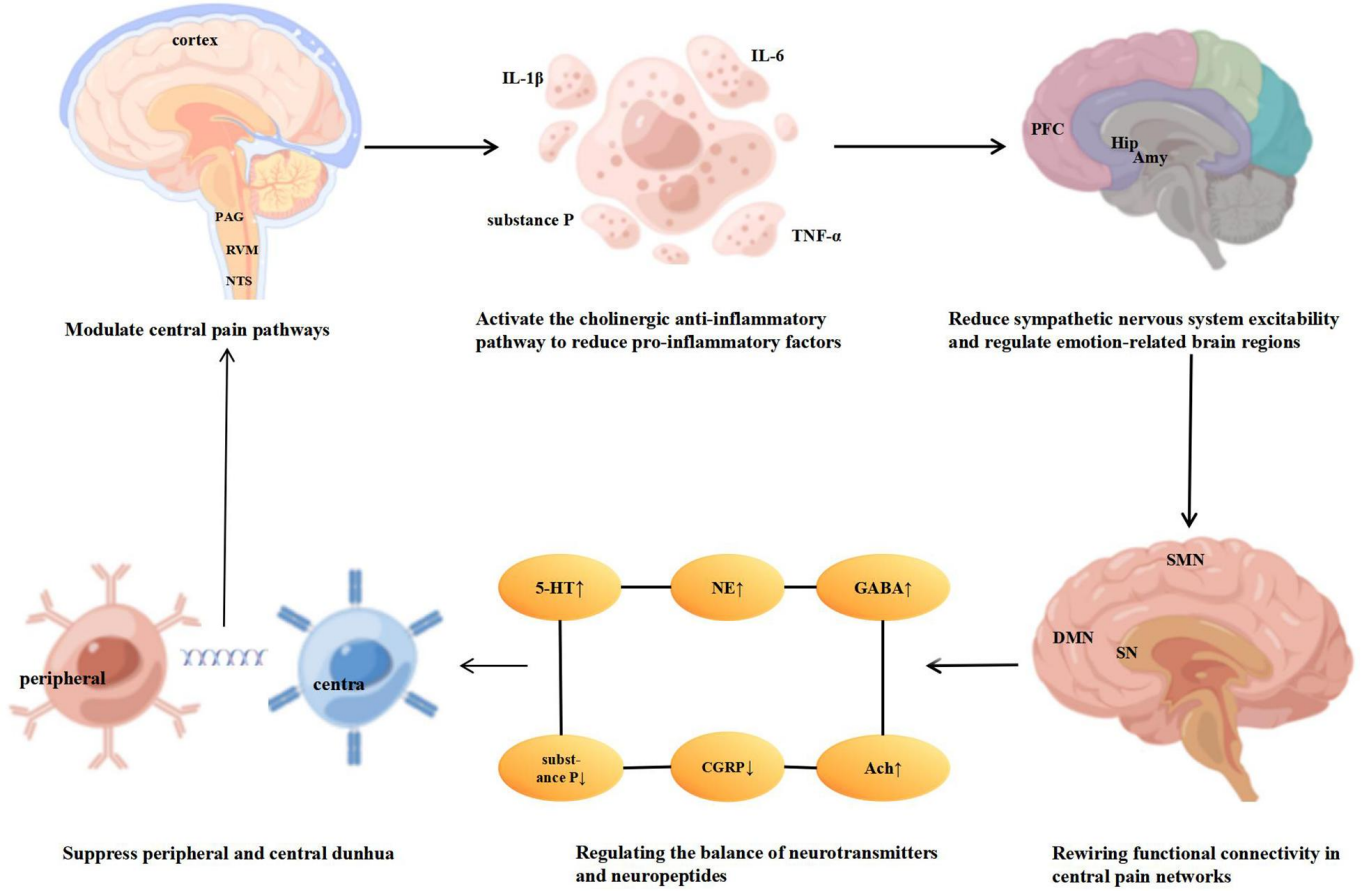
Potential mechanisms of action of taVNS in chronic pain management. Amy, amygdala; DMN, default mode network; Hip, hippocampus; NTS, nucleus tractus solitarius; PAG, periaqueductal gray; PFC, prefrontal cortex; RVM, rostral ventromedial medulla; SMN, somatosensory motor network; SN, salience network.

TaVNS demonstrates multi-target analgesic effects in chronic pain management, primarily achieved through six interrelated mechanismsdemonstrates multi-target analgesic effects in chronic pain management, primarily achieved through six interrelated mechanisms—activating central pain modulation pathways, regulating cholinergic anti-inflammatory pathways, modulating autonomic nervous system and emotional brain regions, reshaping functional connectivity within central pain networks, adjusting neurotransmitter and neuropeptide balance, and inhibiting peripheral and central sensitization (Figure 3). These mechanisms collectively exert therapeutic effects on chronic pain by modulating key pathophysiological components of pain transmission. The following sections provide a systematic review of each mechanism based on existing research evidence.

### 5.1 TaVNS activates central pain modulation pathways

Through multi-node, multi-pathway synergistic effects, taVNS extensively modulates the central pain processing network, constituting its core mechanism for analgesic effects (59). fMRI studies confirm that a key target of taVNS is the periaqueductal gray (PAG) in the midbrain—a pivotal hub of the descending pain modulation system (86, 92). For instance, studies indicate that 1 Hz taVNS specifically enhances FC between the PAG and structures such as the medial cingulate cortex, anterior cingulate cortex, and anterior insula. This may represent the neural mechanism underlying the significant therapeutic efficacy of low-frequency stimulation in chronic migraine (61). In patients with chronic low back pain, taVNS has similarly been shown to remodel the PAG network, specifically by enhancing its FC with the amygdala and sensorimotor cortex. This suggests taVNS not only modulates pain intensity but also improves pain-related emotional and sensory dimensions (86).

More critically, the central regulatory effects of taVNS can be traced along the vagus nerve afferent pathway to the brainstem, where it exerts fundamental influence by modulating key neurochemical systems. The solitary tract nucleus, a primary relay station for vagal afferents, receives signals and further projects to the locus coeruleus and nucleus accumbens, thereby regulating noradrenergic and serotonergic systems. Huang et al. (60) found that following taVNS intervention, FC between the NTS and the hippocampus and prefrontal cortex weakened, potentially contributing to the alleviation of pain-related memory and anxiety. Simultaneously, FC between the raphe nucleus and basal ganglia increased, and this enhancement was significantly correlated with a reduction in migraine attack days. This provides compelling imaging evidence that taVNS suppresses pain perception by enhancing the brainstem-striatal serotonergic pathway. In summary, taVNS does not function through a single “analgesic switch,” but rather by precisely modulating the entire “pain matrix” spanning the brainstem, limbic system, and cortex. This restores the imbalanced endogenous pain regulation and reward system functions observed in chronic pain states.

### 5.2 TaVNS modulation of the cholinergic anti-inflammatory pathway

Inflammation serves as the body's core defensive response to injury or infection, characterized pathologically by persistent infiltration of immune cells predominantly comprising monocytes and lymphocytes (18). This process involves complex cascade reactions mediated by multiple substances and is closely associated with the onset and progression of chronic pain (22). A 2021 bibliometric analysis revealed that research on the co-morbidity mechanisms of inflammation and pain has expanded approximately 192-fold over the past four decades, with neuropathic pain emerging as one of the most prominent research directions (23). This reflects the growing significance of this field within global scientific communities and clinical practice.

Among neuroimmunoregulatory strategies for inflammatory pain, taVNS demonstrates significant therapeutic potential by activating cholinergic anti-inflammatory pathways. Aranow et al. (71) conducted a randomized, double-blind, sham-controlled trial in systemic lupus erythematosus patients, demonstrating that just four days of taVNS intervention significantly reduced plasma levels of substance P—a neuropeptide with dual pro-inflammatory and pain-conducting functions. This reduction correlated positively with improvements in patients’ pain scores. Similarly, Bellocchi et al. (75) reported a 17.1% reduction in serum IL-6 levels in systemic sclerosis patients following one month of taVNS treatment, with its dynamic changes synchronized with improvements in pain scores. Collectively, these findings suggest taVNS may exert therapeutic effects in inflammatory pain management by modulating cholinergic anti-inflammatory pathways to suppress the release of key pro-inflammatory mediators like substance P and IL-6.

Beyond effectively regulating peripheral inflammation, taVNS also influences neuroinflammatory processes through central-peripheral synergistic mechanisms, particularly evident in neuropathic pain. Yang et al. (67) further elucidated that taVNS activates α7 nicotinic acetylcholine receptors to inhibit nuclear factor-*κ*B signaling pathway activity, thereby reducing the release of proinflammatory cytokines such as IL-1β and TNF-α, effectively alleviating chemotherapy-induced peripheral neuropathy-related pain. Although plasma inflammatory cytokine levels showed no significant changes in this study, the reduction in pain intensity and overall improvement in sleep and quality of life suggest that taVNS may achieve systemic regulation and symptom relief in complex pain states through multi-level, multi-pathway neuro-immune interaction mechanisms.

### 5.3 TaVNS modulates the autonomic nervous system and emotional brain regions

From the perspective of autonomic regulation, taVNS effectively enhances parasympathetic activity and suppresses excessive sympathetic excitation by stimulating the vagus nerve branches in the ear, thereby restoring dynamic equilibrium in the autonomic nervous system (93). Studies indicate that taVNS significantly improves clinical symptoms closely related to autonomic function, particularly sleep disorders (62, 88, 90). For instance, in migraine patients, taVNS treatment led to a significant 21.2% reduction in Pittsburgh Sleep Quality Index (PSQI) scores, with improved sleep quality showing a clear correlation to reduced migraine attack frequency (62). In fibromyalgia patients, taVNS has also been demonstrated to improve sleep architecture, with the degree of sleep improvement showing a significant negative correlation to pain score reduction (90). Furthermore, research by Kutlu et al. (88) indicates that taVNS simultaneously modulates autonomic balance, improves emotional states, and promotes overall symptom relief in fibromyalgia. Collectively, these findings suggest taVNS provides a critical physiological basis for alleviating chronic pain and its associated sleep and emotional disturbances by restoring sympathetic-parasympathetic equilibrium.

At the level of brain function regulation, the mechanism of taVNS extends further to emotion- and cognition-related brain regions, particularly through functional modulation of the limbic system and prefrontal cortex, thereby directly alleviating pain-associated emotional disorders (94). Research indicates that taVNS can regulate activity in key limbic system nodes such as the amygdala and hippocampus, while enhancing the function of the dorsolateral prefrontal cortex (dlPFC), which is closely associated with cognitive control and emotional regulation (95). Asmaa et al. (91) found that compared to single interventions, combining taVNS with pain neuroscience education more effectively enhanced dlPFC activity and yielded greater clinical benefits, fully demonstrating taVNS's potential in regulating the prefrontal-limbic neural circuit. Thus, taVNS not only maintains homeostasis through peripheral autonomic pathways but also modulates emotional brain regions via central mechanisms, establishing a dual pathway for treating chronic pain and its emotional comorbidities.

### 5.4 TaVNS remodels functional connectivity in central pain networks

TaVNS effectively improves abnormal pain processing by modulating FC within central pain-related networks. Multiple fMRI studies confirm that taVNS regulates neural activity at key nodes within the default mode network (DMN), sensorimotor network (SMN), and salience network (SN). For instance, Luo et al. (59) observed that a single taVNS intervention in patients with migraine without aura significantly reduced FC between the left amygdala and core DMN regions like the posterior cingulate cortex, while simultaneously attenuating abnormal FC with SMN regions such as the postcentral gyrus. This rapidly alleviated acute-phase pain processing abnormalities. Feng et al. (62) further demonstrated that after 4 weeks of taVNS treatment, amplitude of low frequency fluctuation values in the bilateral anterior cingulate cortex—a key DMN node—significantly decreased, with the reduction positively correlated to reduced migraine attack frequency. This suggests taVNS may restore normal DMN regulation by suppressing its hyperactivity.

Beyond regulating the DMN and SMN, taVNS significantly influences the salience network, which is closely associated with pain emotion and sensory integration. Cai et al. (81) combined taVNS with electroencephalogram studies in patients with persistent abdominal pain, revealing that taVNS markedly reduced functional connectivity strength in the thalamic-insular region within the delta band. This change effectively predicted improvements in patients’ pain scores. This finding suggests that taVNS may alleviate abnormal sensory integration processes in chronic pain by modulating the synchrony of high-frequency oscillations within the sensorimotor and salience networks. Thus, taVNS reshapes the functional architecture of central pain pathways through multi-network synergistic effects, providing robust imaging evidence for neuromodulatory therapies in chronic pain.

### 5.5 TaVNS regulates neurotransmitter and neuropeptide balance

The development of chronic pain is closely associated with an imbalance between excitatory and inhibitory signals in the central and peripheral nervous systems (20). Under physiological conditions, inhibitory neurotransmitters such as serotonin, norepinephrine, and GABA form the basis of endogenous analgesia (29). In contrast, nociceptive neuropeptides like substance P act as excitatory modulators, promoting pain sensitization through mechanisms including enhanced synaptic transmission and induction of neurogenic inflammation (21). Therefore, restoring neurochemical equilibrium is a key strategy for intervening in the vicious cycle of chronic pain.

Aranow et al. (71) demonstrated that taVNS significantly reduced plasma substance P levels in systemic lupus erythematosus patients, with this change positively correlated with improved pain visual analog scale scores. This suggests taVNS may attenuate substance P's pro-pain role in central and peripheral pain transmission by inhibiting its release. Shi et al. (80) further discovered in patients with constipation-predominant irritable bowel syndrome that taVNS not only promotes colonic acetylcholine release and enhances vagal activity but also inhibits P substance release from intestinal nerve endings, thereby alleviating pain transmission in functional abdominal pain. Collectively, these studies reveal taVNS's pivotal role in pain regulation by modulating the dynamic equilibrium of neuropeptides and neurotransmitters. They provide crucial experimental evidence for understanding taVNS's function within the neuro-immune-pain axis and establish a mechanistic foundation for its clinical application in chronic pain management.

### 5.6 TaVNS inhibits peripheral and central sensitization

The core pathophysiological mechanism of chronic pain stems from a vicious cycle involving peripheral and central sensitization (2). Peripheral sensitization begins with local inflammation or nerve injury, leading to increased excitability and lowered thresholds in nociceptors, resulting in pain hypersensitivity (18). Persistent peripheral injury signals further induce central sensitization, manifested as plastic changes in spinal and cerebral pain pathways. Excessive glutamatergic signaling expands neuronal response fields, while the descending inhibitory system, which centers on the locus coeruleus and raphe nuclei, weakens. This ultimately amplifies and generalizes pain signals at the central level (19, 24).

Multiple clinical studies demonstrate that taVNS effectively suppresses peripheral and central sensitization processes through multi-level regulation of pain transmission pathways. At the peripheral level, Bellocchi et al. (75) found taVNS significantly increased the mechanical pain threshold in systemic sclerosis patients, accompanied by decreased serum IL-6 levels, suggesting it alleviates peripheral nerve terminal sensitization via anti-inflammatory mechanisms. At the central level, taVNS suppresses abnormal neuronal excitability by modulating functional connectivity in the brainstem and higher cortical regions. Huang et al. (60) found taVNS effectively suppressed abnormal discharges in the trigeminal spinal nucleus of migraine patients, improving sensitivity to light and sound stimuli. This effect was closely associated with enhanced functional connectivity between the nucleus accumbens and the putamen. Furthermore, Figueiredo et al. (85) observed in chronic low back pain studies that taVNS reduces excessive excitability in spinal dorsal horn neurons, with this effect positively correlated with enhanced FC along the locus coeruleus-nucleus accumbens pathway. These findings systematically elucidate the comprehensive analgesic mechanism of taVNS from peripheral to central pathways.

## 6 Problems and future directions

### 6.1 Standardization research requires further enhancement

Currently, taVNS research lacks unified standards in several critical aspects, limiting the reliability and comparability of its results. Regarding treatment parameters, existing studies predominantly employ different frequencies such as 1 Hz, 20 Hz, or 25 Hz, with no consensus established. Future research should systematically compare the dose-response relationships among different parameters to determine the optimal stimulation protocol. Regarding stimulation site selection, the relative merits of the left concha versus bilateral stimulation remain unclear, necessitating standardized protocols and controlled studies. For control group design, common sham sites include the earlobe or non-innervated areas of the auricle. However, the simulation effectiveness and blinding reliability of these sites require further validation and standardization. Additionally, sample sizes are generally small, and follow-up periods are short. Existing clinical studies predominantly employ 4-week treatment cycles, lacking long-term follow-up data. Future research should prioritize large-scale, multicenter, and extended-duration studies to clarify the long-term efficacy and stability of taVNS.

### 6.2 Insufficient depth in mechanism research

Currently, the precise analgesic mechanism of taVNS remains incompletely elucidated. Existing research exhibits multiple limitations, such as: (1) unclear characterization of the specific neural circuits mediating analgesic effects; (2) lack of direct evidence for the dynamic processes of neuroimmune interactions; (3) insufficient exploration of the mechanistic heterogeneity across chronic pain conditions of different etiologies. Future research urgently requires integrating techniques such as multimodal neuroimaging, molecular imaging, and biomarker analysis to establish more robust connections between macro-network and micro-molecular levels, thereby systematically elucidating the analgesic mechanisms of taVNS.

### 6.3 Personalized and precision treatment strategies require further development

Currently, the exploration of taVNS in the field of personalized and precision medicine remains in its early stages, directly limiting the optimization of its clinical efficacy and widespread application. First, there is a lack of stable, universally applicable biomarkers for predicting treatment outcomes. Although a few studies have attempted to correlate changes in brain network FC revealed by fMRI with clinical improvement (61), these imaging markers remain unstable and are costly to obtain, making them impractical as routine predictive tools. Recent research has identified several promising candidate biomarkers for taVNS response. For example, HRV, reflecting autonomic modulation, may predict pain reduction in fibromyalgia patients (90). Inflammatory markers such as IL-6 and substance P decrease after taVNS, correlating with clinical improvements in autoimmune-related pain (71, 75). Neuroimaging markers, including resting-state functional connectivity between the periaqueductal gray and limbic regions, also show predictive value for migraine outcomes (61). Electroencephalogram (EEG) signatures—especially theta and alpha band power changes—have been linked to acute analgesic responses in abdominal pain (81). While promising, the specificity, reproducibility, and clinical utility of these biomarkers require further validation in large prospective cohorts. Second, stimulation parameter settings lack individualized theoretical guidance. Current protocols predominantly employ uniform frequencies and fixed intensity adjustments based on sensory thresholds, failing to account for patients’ distinct pain etiologies, pathophysiological underpinnings, or specific clinical manifestations. Future research urgently requires the deep integration of multi-omics data, neurophysiological indicators, and clinical phenotypes. Leveraging advanced algorithms such as machine learning, decision support systems capable of precisely guiding patient selection, parameter optimization, and treatment plan formulation must be developed to ultimately maximize the clinical value of taVNS.

### 6.4 Further strengthening is needed to promote combination therapy

Although multiple studies have preliminarily confirmed the good safety and tolerability of taVNS as a monotherapy, its synergistic effects in combination therapy remain understudied. Currently, only a few studies have attempted to combine taVNS with other non-pharmacological therapies. For example, Abdel-Baset et al. (91) demonstrated that taVNS combined with PNE outperformed monotherapy in improving pain and mood in fibromyalgia patients. Conversely, Kutlu et al. (88) found that adding taVNS to structured exercise did not yield significant additional benefits. These inconsistent findings suggest that the efficacy of taVNS in combination therapy may be influenced by multiple factors, including disease type, treatment parameters, co-administered interventions, and patient characteristics. Future research requires well-designed, adequately powered randomized controlled trials to explore optimized combination regimens of taVNS with pharmacotherapy, physical therapy, psychological interventions, and other modalities. This will clarify synergistic mechanisms and establish personalized combination treatment strategies to enhance the overall efficacy and clinical applicability of taVNS in managing complex chronic pain.

## 7 Limitations of this review

First, the chronic pain types covered in this paper exhibit high diversity, including neuropathic pain, autoimmune-related pain, gastrointestinal pain, and musculoskeletal pain. While this reflects the broad application potential of taVNS, the fundamentally different pathophysiological mechanisms underlying these pain types result in varying intervention response characteristics. This poses challenges for direct comparisons and rational interpretations of disparate research findings. Second, the number of high-quality studies for each pain subtype remains limited, with significant clinical heterogeneity observed in study populations, stimulation parameters, and efficacy assessment metrics. Consequently, this review primarily employs qualitative synthesis and narrative analysis methods, precluding quantitative meta-analysis. This substantially limits robust statistical inference regarding the overall effect size of taVNS and hinders the conduct of persuasive subgroup analyses. Finally, most explanations of the mechanism of action in the literature are based on correlational evidence observed across different studies, lacking direct experimental validation of causal pathways. Consequently, the explanatory power of these mechanisms requires further consolidation through future research.

## 8 Conclusion

TaVNS emerges as an innovative non-invasive neuromodulation technique demonstrating multifaceted therapeutic value in chronic pain management. It exhibits significant efficacy across diverse chronic pain conditions including migraine, neuropathic pain, autoimmune diseases, and gastrointestinal dysfunction. Beyond alleviating pain intensity, taVNS concurrently improves mood disorders and sleep disturbances. The analgesic effects of taVNS are primarily achieved through six interconnected mechanisms: activating central descending pain control pathways, modulating cholinergic anti-inflammatory pathways, balancing autonomic nervous system function, remodeling functional connectivity within brain networks, regulating neurotransmitter and neuropeptide balance, and inhibiting peripheral and central sensitization processes. Despite current challenges such as insufficient treatment standardization and limited mechanistic understanding, taVNS offers an innovative therapeutic strategy for chronic pain patients. Its favorable safety profile and multi-targeted regulatory advantages enable a shift from mere symptom control to neural functional modulation. Future large-scale clinical trials and multidisciplinary collaboration are needed to optimize treatment protocols and advance its precise application within comprehensive pain management systems.

## References

[1] LuoGibbs D McGahan BG Ropper AE Back pain: differential diagnosis and management. Neurol Clin. (2023) 41(1):61–76. 10.1016/j.ncl.2022.07.002 36400559

[2] Cohen SP Vase L Hooten WM. Chronic pain: an update on burden, best practices, and new advances. Lancet. (2021) 397(10289):2082–2097. 10.1016/S0140-6736(21)00393-7 34062143

[3] Bell T Franz CE Kremen WS. Persistence of pain and cognitive impairment in older adults. J Am Geriatr Soc. (2022) 70(2):449–458. 10.1111/jgs.17542 34741304 PMC8821128

[4] Vickers AJ Vertosick EA Lewith G Acupuncture for chronic pain: update of an individual patient data meta-analysis. J Pain. (2018) 19(5):455–474. 10.1016/j.jpain.2017.11.005 29198932 PMC5927830

[5] Layne-Stuart CM Carpenter AL. Chronic pain considerations in patients with cardiovascular disease. Anesthesiol Clin. (2022) 40(4):791–802. 10.1016/j.anclin.2022.08.018 36328629

[6] Wang D. Opioid medications in the management of chronic abdominal pain. Curr Pain Headache Rep. (2017) 21(9):40. 10.1007/s11916-017-0640-x 28791598

[7] Tobin DG Lockwood MB Kimmel PL Opioids for chronic pain management in patients with dialysis-dependent kidney failure. Nat Rev Nephrol. (2022) 18(2):113–128. 10.1038/s41581-021-00484-6 34621058 PMC8792317

[8] Shi Y Wu W. Multimodal non-invasive non-pharmacological therapies for chronic pain: mechanisms and progress. BMC Med. (2023) 21(1):372. 10.1186/s12916-023-03076-2 37775758 PMC10542257

[9] Adams N Poole H Richardson C. Psychological approaches to chronic pain management: part 1. J Clin Nurs. (2006) 15(3):290–300. 10.1111/j.1365-2702.2006.01304.x 16466478

[10] Guven Kose S Kose HC Celikel F Chronic pain: an update of clinical practices and advances in chronic pain management. Eurasian J Med. (2022) 54(Suppl1):57–61. 10.5152/eurasianjmed.2022.22307 36655446 PMC11163351

[11] Dubin AE Patapoutian A. Nociceptors: the sensors of the pain pathway. J Clin Invest. (2010) 120(11):3760–3772. 10.1172/JCI42843 21041958 PMC2964977

[12] Basbaum AI Bautista DM Scherrer G Cellular and molecular mechanisms of pain. Cell. (2009) 139(2):267–284. 10.1016/j.cell.2009.09.028 19837031 PMC2852643

[13] Finnerup NB Kuner R Jensen TS. Neuropathic pain: from mechanisms to treatment. Physiol Rev. (2021) 101(1):259–301. 10.1152/physrev.00045.2019 32584191

[14] Gilbert JE Zhang T Esteller R Network model of nociceptive processing in the superficial spinal dorsal horn reveals mechanisms of hyperalgesia, allodynia, and spinal cord stimulation. J Neurophysiol. (2023) 130(5):1103–1117. 10.1152/jn.00186.2023 37727912

[15] Todd AJ. Neuronal circuitry for pain processing in the dorsal horn. Nat Rev Neurosci. (2010) 11(12):823–836. 10.1038/nrn2947 21068766 PMC3277941

[16] Melzack R Wall PD. Pain mechanisms: a new theory. Science. (1965) 150(3699):971–979. 10.1126/science.150.3699.971 5320816

[17] Yao D Chen Y Chen G. The role of pain modulation pathway and related brain regions in pain. Rev Neurosci. (2023) 34(8):899–914. 10.1515/revneuro-2023-0037 37288945

[18] Szok D Tajti J Nyári A Therapeutic approaches for peripheral and central neuropathic pain. Behav Neurol. (2019) 2019:8685954. 10.1155/2019/8685954 31871494 PMC6906810

[19] Fitzgerald CT Carter LP. Possible role for glutamic acid decarboxylase in fibromyalgia symptoms: a conceptual model for chronic pain. Med Hypotheses. (2011) 77(3):409–415. 10.1016/j.mehy.2011.05.031 21684692

[20] Ji RR Nackley A Huh Y Neuroinflammation and central sensitization in chronic and widespread pain. Anesthesiology. (2018) 129(2):343–366. 10.1097/ALN.0000000000002130 29462012 PMC6051899

[21] Humes C Sic A Knezevic NN. Substance p’s impact on chronic pain and psychiatric conditions-A narrative review. Int J Mol Sci. (2024) 25(11):5905. 10.3390/ijms25115905 38892091 PMC11172719

[22] Fang XX Zhai MN Zhu M Inflammation in pathogenesis of chronic pain: foe and friend. Mol Pain. (2023) 19:17448069231178176. 10.1177/17448069231178176 37220667 PMC10214073

[23] Xiong HY Zhang ZJ Wang XQ. Bibliometric analysis of research on the comorbidity of pain and inflammation. Pain Res Manag. (2021) 2021:6655211. 10.1155/2021/6655211 33680225 PMC7904349

[24] Fillingim RB Loeser JD Baron R Assessment of chronic pain: domains, methods, and mechanisms. J Pain. (2016) 17(9 Suppl):T10–T20. 10.1016/j.jpain.2015.08.010 27586827 PMC5010652

[25] Heinricher MM Tavares I Leith JL Descending control of nociception: specificity, recruitment and plasticity. Brain Res Rev. (2009) 60(1):214–225. 10.1016/j.brainresrev.2008.12.009 19146877 PMC2894733

[26] Apkarian AV Bushnell MC Treede RD Human brain mechanisms of pain perception and regulation in health and disease. Eur J Pain. (2005) 9(4):463–484. 10.1016/j.ejpain.2004.11.001 15979027

[27] Cheng K Martin LF Slepian MJ Mechanisms and pathways of pain photobiomodulation: a narrative review. J Pain. (2021) 22(7):763–777. 10.1016/j.jpain.2021.02.005 33636371 PMC8277709

[28] Pak DJ Yong RJ Kaye AD Chronification of pain: mechanisms, current understanding, and clinical implications. Curr Pain Headache Rep. (2018) 22(2):9. 10.1007/s11916-018-0666-8 29404791

[29] Hooten WM. Chronic pain and mental health disorders: shared neural mechanisms, epidemiology, and treatment. Mayo Clin Proc. (2016) 91(7):955–970. 10.1016/j.mayocp.2016.04.029 27344405

[30] Gevers-Montoro C Provencher B Descarreaux M Neurophysiological mechanisms of chiropractic spinal manipulation for spine pain. Eur J Pain. (2021) 25(7):1429–1448. 10.1002/ejp.1773 33786932

[31] Willis WD Westlund KN. Neuroanatomy of the pain system and of the pathways that modulate pain. J Clin Neurophysiol. (1997) 14(1):2–31. 10.1097/00004691-199701000-00002 9013357 PMC7859971

[32] Duncan GH Bushnell MC Talbot JD Pain and activation in the thalamus. Trends Neurosci. (1992) 15(7):252–253. 10.1016/0166-2236(92)90062-d 1381119

[33] Tracey I Mantyh PW. The cerebral signature for pain perception and its modulation. Neuron. (2007) 55(3):377–391. 10.1016/j.neuron.2007.07.012 17678852

[34] Ong WY Stohler CS Herr DR. Role of the prefrontal cortex in pain processing. Mol Neurobiol. (2019) 56(2):1137–1166. 10.1007/s12035-018-1130-9 29876878 PMC6400876

[35] Volcheck MM Graham SM Fleming KC Central sensitization, chronic pain, and other symptoms: better understanding, better management. Cleve Clin J Med. (2023) 90(4):245–254. 10.3949/ccjm.90a.22019 37011956

[36] Wang Y Li SY Wang D Transcutaneous auricular vagus nerve stimulation: from concept to application. Neurosci Bull. (2021) 37(6):853–862. 10.1007/s12264-020-00619-y 33355897 PMC8192665

[37] Alimi D Chelly JE. New universal Nomenclature in auriculotherapy. J Altern Complement Med. (2018) 24(1):7–14. 10.1089/acm.2016.0351 28832182

[38] Butt MF Albusoda A Farmer AD The anatomical basis for transcutaneous auricular vagus nerve stimulation. J Anat. (2020) 236(4):588–611. 10.1111/joa.13122 31742681 PMC7083568

[39] Yap JYY Keatch C Lambert E Critical review of transcutaneous Vagus nerve stimulation: challenges for translation to clinical practice. Front Neurosci. (2020) 14:284. 10.3389/fnins.2020.00284 32410932 PMC7199464

[40] Zhu J Wang J Xu C The functional connectivity study on the brainstem-cortical/subcortical structures in responders following cervical vagus nerve stimulation. Int J Dev Neurosci. (2020) 80(8):679–686. 10.1002/jdn.10064 32931055

[41] Hilz MJ. Transcutaneous vagus nerve stimulation - A brief introduction and overview. Auton Neurosci. (2022) 243:103038. 10.1016/j.autneu.2022.103038 36201901

[42] Baig SS Kamarova M Ali A Transcutaneous vagus nerve stimulation (tVNS) in stroke: the evidence, challenges and future directions. Auton Neurosci. (2022) 237:102909. 10.1016/j.autneu.2021.102909 34861612

[43] Li TT Wang ZJ Yang SB Transcutaneous electrical stimulation at auricular acupoints innervated by auricular branch of vagus nerve pairing tone for tinnitus: study protocol for a randomized controlled clinical trial. Trials. (2015) 16:101. 10.1186/s13063-015-0630-4 25872506 PMC4384366

[44] Johnson MI Paley CA Jones G Mulvey MR Wittkopf PG. Efficacy and safety of transcutaneous electrical nerve stimulation (TENS) for acute and chronic pain in adults: a systematic review and meta-analysis of 381 studies (the meta-TENS study). BMJ Open. (2022) 12(2):e051073. 10.1136/bmjopen-2021-051073 35144946 PMC8845179

[45] Pérez-Carbonell L Faulkner H Higgins S Vagus nerve stimulation for drug-resistant epilepsy. Pract Neurol. (2020) 20(3):189–198. 10.1136/practneurol-2019-002210 31892545

[46] Chrastina J Dolezalova I Novak Z Pregnancy outcomes in refractory epilepsy patients with Vagus nerve stimulation: long-term single-center experience. J Neurol Surg A Cent Eur Neurosurg. (2022) 83(3):259–264. 10.1055/s-0041-1730966 34496415

[47] González HFJ Yengo-Kahn A Englot DJ. Vagus nerve stimulation for the treatment of epilepsy. Neurosurg Clin N Am. (2019) 30(2):219–230. 10.1016/j.nec.2018.12.005 30898273 PMC6432928

[48] Thompson SL O'Leary GH Austelle CW A review of parameter settings for invasive and non-invasive Vagus nerve stimulation (VNS) applied in neurological and psychiatric disorders. Front Neurosci. (2021) 15:709436. 10.3389/fnins.2021.709436 34326720 PMC8313807

[49] Yakunina N Kim SS Nam EC. Optimization of transcutaneous Vagus nerve stimulation using functional MRI. Neuromodulation. (2017) 20(3):290–300. 10.1111/ner.12541 27898202

[50] Sclocco R Garcia RG Kettner NW Stimulus frequency modulates brainstem response to respiratory-gated transcutaneous auricular vagus nerve stimulation. Brain Stimul. (2020) 13(4):970–978. 10.1016/j.brs.2020.03.011 32380448 PMC7931850

[51] Schmidt-Wilcke T. Neuroimaging of chronic pain. Best Pract Res Clin Rheumatol. (2015) 29(1):29–41. 10.1016/j.berh.2015.04.030 26266997

[52] Redgrave J Day D Leung H Safety and tolerability of transcutaneous Vagus nerve stimulation in humans; a systematic review. Brain Stimul. (2018) 11(6):1225–1238. 10.1016/j.brs.2018.08.010 30217648

[53] Costa V Gianlorenço AC Andrade MF Transcutaneous vagus nerve stimulation effects on chronic pain: systematic review and meta-analysis. Pain Rep. (2024) 9(5):e1171. 10.1097/PR9.0000000000001171 39131814 PMC11309651

[54] Austelle CW O'Leary GH Thompson S A comprehensive review of Vagus nerve stimulation for depression. Neuromodulation. (2022) 25(3):309–315. 10.1111/ner.13528 35396067 PMC8898319

[55] Ferrari MD Goadsby PJ Burstein R Migraine. Nat Rev Dis Primers. (2022) 8(1):2. 10.1038/s41572-021-00328-4 35027572

[56] Stovner LJ Hagen K Linde M The global prevalence of headache: an update, with analysis of the influences of methodological factors on prevalence estimates. J Headache Pain. (2022) 23(1):34. 10.1186/s10194-022-01402-2 35410119 PMC9004186

[57] Straube A Ellrich J Eren O Treatment of chronic migraine with transcutaneous stimulation of the auricular branch of the vagal nerve (auricular t-VNS): a randomized, monocentric clinical trial. J Headache Pain. (2015) 16:543. 10.1186/s10194-015-0543-3 26156114 PMC4496420

[58] Zhang Y Huang Y Li H Transcutaneous auricular vagus nerve stimulation (taVNS) for migraine: an fMRI study. Reg Anesth Pain Med. (2021) 46(2):145–150. 10.1136/rapm-2020-102088 33262253

[59] Luo W Zhang Y Yan Z The instant effects of continuous transcutaneous auricular Vagus nerve stimulation at acupoints on the functional connectivity of amygdala in migraine without aura: a preliminary study. Neural Plast. (2020) 2020:8870589. 10.1155/2020/8870589 33381165 PMC7759401

[60] Huang Y Zhang Y Hodges S The modulation effects of repeated transcutaneous auricular vagus nerve stimulation on the functional connectivity of key brainstem regions along the vagus nerve pathway in migraine patients. Front Mol Neurosci. (2023) 16:1160006. 10.3389/fnmol.2023.1160006 37333617 PMC10275573

[61] Cao J Zhang Y Li H Different modulation effects of 1 hz and 20 hz transcutaneous auricular vagus nerve stimulation on the functional connectivity of the periaqueductal gray in patients with migraine. J Transl Med. (2021) 19(1):354. 10.1186/s12967-021-03024-9 34404427 PMC8371886

[62] Feng M Zhang Y Wen Z Early fractional amplitude of low frequency fluctuation can predict the efficacy of transcutaneous auricular Vagus nerve stimulation treatment for migraine without aura. Front Mol Neurosci. (2022) 15:778139. 10.3389/fnmol.2022.778139 35283732 PMC8908103

[63] Rao Y Liu W Zhu Y Altered functional brain network patterns in patients with migraine without aura after transcutaneous auricular vagus nerve stimulation. Sci Rep. (2023) 13(1):9604. 10.1038/s41598-023-36437-1 37311825 PMC10264378

[64] Yeh ML Liao RW Yeh PH Acupuncture-related interventions improve chemotherapy-induced peripheral neuropathy: a systematic review and network meta-analysis. BMC Complement Med Ther. (2024) 24(1):310. 10.1186/s12906-024-04603-1 39160496 PMC11334450

[65] Molinares D Kurtevski S Zhu Y. Chemotherapy-Induced peripheral neuropathy: diagnosis, agents, general clinical presentation, and treatments. Curr Oncol Rep. (2023) 25(11):1227–1235. 10.1007/s11912-023-01449-7 37702983

[66] Loprinzi CL Lacchetti C Bleeker J Prevention and management of chemotherapy-induced peripheral neuropathy in survivors of adult cancers: aSCO guideline update. J Clin Oncol. (2020) 38(28):3325–3348. 10.1200/JCO.20.01399 32663120

[67] Yang Y Zhang R Zhong Z Efficacy of transauricular vagus nerve stimulation for the treatment of chemotherapy-induced painful peripheral neuropathy: a randomized controlled exploratory study. Neurol Sci. (2024) 45(5):2289–2300. 10.1007/s10072-023-07229-2 38063922

[68] Hoi A Igel T Mok CC Systemic lupus erythematosus. Lancet. (2024) 403(10441):2326–2338. 10.1016/S0140-6736(24)00398-2=-64342134 38642569 10.1016/S0140-6736(24)00398-2

[69] Lazar S Kahlenberg JM. Systemic lupus erythematosus: new diagnostic and therapeutic approaches. Annu Rev Med. (2023) 74:339–352. 10.1146/annurev-med-043021-032611 35804480

[70] Koopman FA Chavan SS Miljko S Vagus nerve stimulation inhibits cytokine production and attenuates disease severity in rheumatoid arthritis. Proc Natl Acad Sci U S A. (2016) 113(29):8284–8289. 10.1073/pnas.1605635113 27382171 PMC4961187

[71] Aranow C Atish-Fregoso Y Lesser M Transcutaneous auricular vagus nerve stimulation reduces pain and fatigue in patients with systemic lupus erythematosus: a randomised, double-blind, sham-controlled pilot trial. Ann Rheum Dis. (2021) 80(2):203–208. 10.1136/annrheumdis-2020-217872 33144299

[72] Volkmann ER Andréasson K Smith V. Systemic sclerosis. Lancet. (2023) 401(10373):304–318. 10.1016/S0140-6736(22)01692-0 36442487 PMC9892343

[73] Carandina A Bellocchi C Dias Rodrigues G Cardiovascular autonomic control, sleep and health related quality of life in systemic sclerosis. Int J Environ Res Public Health. (2021) 18(5):2276. 10.3390/ijerph18052276 33668942 PMC7956693

[74] Jaeger VK Distler O Maurer B Functional disability and its predictors in systemic sclerosis: a study from the DeSScipher project within the EUSTAR group. Rheumatology (Oxford). (2018) 57(3):441–450. 10.1093/rheumatology/kex182 28499034

[75] Bellocchi C Carandina A Della Torre A Transcutaneous auricular branch vagal nerve stimulation as a non-invasive add-on therapeutic approach for pain in systemic sclerosis. RMD Open. (2023) 9(3):e003265. 10.1136/rmdopen-2023-003265 37536947 PMC10401218

[76] Huang KY Wang FY Lv M Irritable bowel syndrome: epidemiology, overlap disorders, pathophysiology and treatment. World J Gastroenterol. (2023) 29(26):4120–4135. 10.3748/wjg.v29.i26.4120 37475846 PMC10354571

[77] Ford AC Sperber AD Corsetti M Irritable bowel syndrome. Lancet. (2020) 396(10263):1675–1688. 10.1016/S0140-6736(20)31548-8 33049223

[78] Chey WD Kurlander J Eswaran S. Irritable bowel syndrome: a clinical review. JAMA. (2015) 313(9):949–958. 10.1001/jama.2015.0954 25734736

[79] Sebastián Domingo JJ. Irritable bowel syndrome. Síndrome del intestino irritable. Med Clin (Barc). (2022) 158(2):76–81. 10.1016/j.medcli.2021.04.029 34238582

[80] Shi X Hu Y Zhang B Ameliorating effects and mechanisms of transcutaneous auricular vagal nerve stimulation on abdominal pain and constipation. JCI Insight. (2021) 6(14):e150052. 10.1172/jci.insight.150052 34138761 PMC8410029

[81] Cai S Li Q Liu L Predictive value of acute neuroplastic response to taVNS in treatment outcome in persistent abdominal pain: a concurrent taVNS-EEG trial. Brain Stimul. (2025) 18(5):1511–1513. 10.1016/j.brs.2025.08.009 40812619

[82] Zhou T Salman D McGregor AH. Recent clinical practice guidelines for the management of low back pain: a global comparison. BMC Musculoskelet Disord. (2024) 25(1):344. 10.1186/s12891-024-07468-0 38693474 PMC11061926

[83] Thiveos L Kent P Pocovi NC Cognitive functional therapy for chronic low back pain: a systematic review and meta-analysis. Phys Ther. (2024) 104(12):pzae128. 10.1093/ptj/pzae128 39236249 PMC11649759

[84] Diamond S Borenstein D. Chronic low back pain in a working-age adult. Best Pract Res Clin Rheumatol. (2006) 20(4):707–720. 10.1016/j.berh.2006.04.002 16979534

[85] Tavares-Figueiredo I Pers YM Duflos C Effect of transcutaneous auricular vagus nerve stimulation in chronic low back pain: a pilot study. J Clin Med. (2024) 13(24):7601. 10.3390/jcm13247601 39768526 PMC11677670

[86] Li T Wu Y Li Y Transcutaneous auricular nerve stimulation modulates the functional connectivity of the descending pain modulation system and reward network in patients with chronic low back pain. Neurotherapeutics. (2025) 22(5):e00611. 10.1016/j.neurot.2025.e00611 40461351 PMC12491802

[87] Berwick R Barker C Goebel A The diagnosis of fibromyalgia syndrome. Clin Med (Lond). (2022) 22(6):570–574. 10.7861/clinmed.2022-0402 36427885 PMC9761415

[88] Kutlu N Özden AV Alptekin HK The impact of auricular vagus nerve stimulation on pain and life quality in patients with fibromyalgia syndrome. Biomed Res Int. (2020) 2020:8656218. 10.1155/2020/8656218 32190684 PMC7071794

[89] Favretti M Iannuccelli C Di Franco M. Pain biomarkers in fibromyalgia syndrome: current understanding and future directions. Int J Mol Sci. (2023) 24(13):10443. 10.3390/ijms241310443 37445618 PMC10341963

[90] Paccione CE Stubhaug A Diep LM Meditative-based diaphragmatic breathing vs. vagus nerve stimulation in the treatment of fibromyalgia-A randomized controlled trial: body vs. machine. Front Neurol. (2022) 13:1030927. 10.3389/fneur.2022.1030927 36438970 PMC9687386

[91] Abdel-Baset AM Abdellatif MA Ahmed HHS Pain neuroscience education versus transcutaneous vagal nerve stimulation in the management of patients with fibromyalgia. Egyptian Rheumatologist. (2023) 45(3):191–195. 10.1016/j.ejr.2023.03.001

[92] Borgmann D Rigoux L Kuzmanovic B Technical note: modulation of fMRI brainstem responses by transcutaneous vagus nerve stimulation. Neuroimage. (2021) 244:118566. 10.1016/j.neuroimage.2021.118566 34509623

[93] Wang W Wang M Ma C Transcutaneous auricular vagus nerve stimulation attenuates stroke-heart syndrome: the role of parasympathetic activity. Exp Neurol. (2025) 385:115094. 10.1016/j.expneurol.2024.115094 39637965

[94] Sun J Sun K Chen L A predictive study of the efficacy of transcutaneous auricular vagus nerve stimulation in the treatment of major depressive disorder: an fMRI-based machine learning analysis. Asian J Psychiatr. (2024) 98:104079. 10.1016/j.ajp.2024.104079 38838458

[95] Liu CH Yang MH Zhang GZ Neural networks and the anti-inflammatory effect of transcutaneous auricular vagus nerve stimulation in depression. J Neuroinflammation. (2020) 17(1):54. 10.1186/s12974-020-01732-5 32050990 PMC7017619

